# Effects of a multifaceted intervention QI program to improve ICU performance

**DOI:** 10.1186/s12913-018-3648-y

**Published:** 2018-11-07

**Authors:** Anders Ersson, Anders Beckman, Johan Jarl, Jonas Borell

**Affiliations:** 1 Department of Intensive Care and Perioperative medicine, Skåne University Hospital, Malmö, Sweden; 2 Department of Clinical Sciences Malmö, Family Medicine, Lund University, Jan Waldenströms gata 35, 205 02 Malmö, Sweden; 3 Department of Clinical Sciences Malmö, Health Economics, Lund University, Malmö, Sweden; 4 Department of Design Sciences, Faculty of Engineering, Lund University, Lund, Sweden

**Keywords:** ICU, Critical care, LEAN, Quality improvement, Organisation, Outcome, Cost-effectiveness

## Abstract

**Background:**

To benefit from the increasing clinical evidence, organisational changes have been among the main drivers behind the reduction of ICU mortality during the last decade. Increasing demand, costs and complexity, amplifies the need for optimisation of clinical processes and resource utilisation. Thus, multidisciplinary teamwork and critical care processes needs to be adapted to profit from increased availability of human skill and technical resources in a cost-effective manner. Inadequate clinical performance and outcome data compelled us to design a quality improvement project to address current work processes and competence utilisation.

**Methods:**

During revision period, clinical processes, professional performance and clinical competence were targeted using “scientific production management methodology” approach. As part of the project, an intensivist training program was instituted, and full time intensivist coverage was obtained in the process of creating multi-professional teams, composed of certified intensivists, critical care nurses, assistant nurses, physiotherapists and social counsellors. The use of staff resources and clinical work-processes were optimised in accordance with the outcome of a “value stream mapping”. In this process, efforts to enhance the personal dynamics and performance within the teams were paramount. Clinical and economic outcome data were analysed during a seven year follow up period.

**Results:**

• Consecutive reduced overall ICU (24%) and long-term (600 days) mortality. The effect on ICU mortality was especially pronounced in the subgroup of patients > 65 years (30%)

• Consecutive reduced length of stay (43%, septic patients) and time on ventilator (for septic patients and patients > 65 years of age (23 resp.52%).

• Substantial increase in life years gained (13,140 life years) as well as quality-adjusted life-years (9593 QALY: s) over the study period.

• High cost-effectiveness as ICU costs were  reduced while patient outcomes were improved. Disregarding the cost reduction in ICU, the intervention is highly cost effective with cost- effectiveness ratios of (75€/QALY) and (55€ / life year)

**Conclusions:**

We have shown favourable results of a QI project aiming to improve the clinical performance and quality through the development of multi-professional interaction, teamwork and systematic revisions of work processes. The economic evaluation shows that the intervention is highly cost-effective and potentially dominating.

## Background

During the past decades, intensive care has undergone a rapid development, during which technological resources as well as the body of scientific knowledge have grown extensively, making critical care available to an increasing number of patients with high comorbidity and age [1–3]. This growing complexity of critical care, including more pre-emptive activities, will amplify the need for multidisciplinary involvement, and as a consequence cross today’s professional boundaries [3, 4]. However, errors and diversions from Evidence Based Medicine (EBM) guidelines are common and have been shown to significantly contribute to increases in mortality, morbidity, length of stay (LOS) and the consumption of economic resources [5]. Unless clinical critical care and connected processes are adapted to profit from the increased availability of human skill and technical resources, it is unlikely the mere existence of these resources would have any major influence on clinical outcomes [1–4, 6, 7].

Despite encouraging results from pivotal studies on the introductions of new drugs, equipment and therapies, the major steps forward needed to implement new knowledge into clinical practice have frequently been accomplished through campaigns or programs targeting organisational changes [6, 8–14]. To achieve this type of change, a non-hierarchical, clinically oriented and enabling leadership, able to manage change and competing logics, has been recognised as crucial as it facilitates the necessary transformation of the organisational culture and enables staff to participate in the change process [11, 15–23]. This implies adjustments in resource allocation, organisation and training so that adequate competence is accessible both in quality and in sufficient numbers (24 h/7d) in relation to caseload and clinical demands [1, 4, 24].

This paper describes a quality improvement (QI) process in an ICU, highlighting its clinical and economic outcomes. The incentive for the intervention was experienced quality and outcome-related problems, displayed in the national critical care registry. Benchmark data from the registry regarding items such as mortality, long-term outcome, time to interventions, and complications were found unfavourable in comparison with national data. Biannual work environment audits and analysis of unit incident reporting, also showed recurrent evidence of inconsistent compliance with EBM guidelines and a prolonged clinical decision process emanating from organisational and competence issues. Also evident in the reporting was an unfavourable staffing situation with diffuse and informal physician leadership, thus impairing collaboration with experienced nursing staff and supervision of clinical proceedings, all with the potential to affect patient safety.

One of the unit’s main problems in performing team-based EBM care was that the physicians were not a part of the ICU staff and that their staffing and work hours were not compliant with the organisational needs of the unit, thus impairing 24 h/7d care and the execution of multi-professional teamwork. Previously, physician staffing models with a daytime workforce and a reduced night shift staff, consisting of mainly non-intensivists, during off hours has been suggested to be abandoned in favour of a 24 h/7d intensivist supervised care and “high-intensity staffing” organised in multidisciplinary teams [1, 4, 25, 26].

The aim of the intervention was to improve quality and outcome, ability to perform EBM critical care with 24 h/7d capacity and to improve patient safety. To accomplish this, a need for reorganisation of the clinical work and implementation of team-based care were identified as key strategies. This study aims to establish the clinical and economic effects of the organizational intervention.

## Methods

**Fig. 1:**
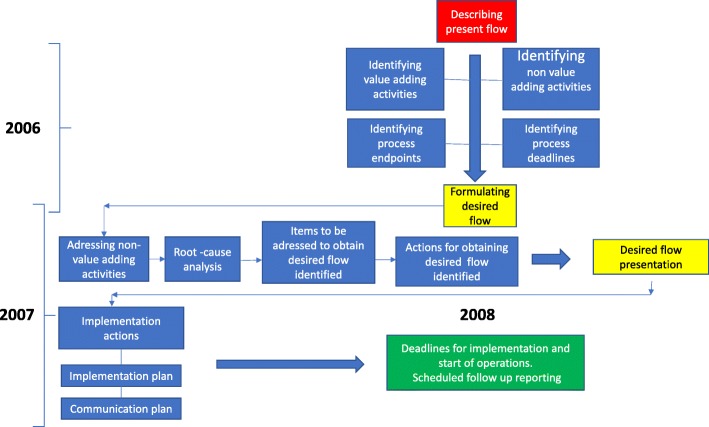
Schematic graphics of the Value Flow Mapping

All clinical activities and organisational structures were analysed and revised during a two-year period (2006–2007) with particular focus on process control. The revision was driven by a designated task force containing representatives from all ICU professions. The items to address emanated from the results produced in multiple “all staff” seminars and subsequent workgroups during 2006–2007 (Table 1). For a thorough description of this process we refer to recent publication (19). During the phase of analysis and revision of the ICU process (2006–2007), no interventions or alterations in the clinical practice or organisation of the ICU were performed. As a result of the preparatory work done during 2006–2007, a revised workflow and organisational change, aiming to secure ability and capacity to deliver EBM critical care 24 h/7d was implemented in the ICU in January 2008. The workflow analysis resulted in a change process that contained the following interventions, schematically described in Fig. 1:

- Synchronisation of clinical work and schedules into a common workflow.
  All clinical activities performed by the different participating professional groups were described and organised in a common coherent workflow. In this flow, identified “waste” in the value stream mapping (VSM) such as waiting times, recurrent errors, equipment failures or incapacities, lack of staff capacity or competence, conflicting demand situations etc. were acted upon and eliminated. All value adding activities, such as hard limits for reporting, round-times, documentation etc. were amplified and timely organised with reference to the agreed process end and time points defined in the VSM.
- The creation and implementation of multi-professional teams
  Instead of different professions working in parallel tracks, all ICU staff were integrated in teams consisting of physicians, nurses, assistant nurses, physiotherapists and social counsellors. To achieve this, working schedules were synchronised for all staff to enable common hand-over procedures and staff continuity. The teams performed as a unit and uniform procedures for team performance such as “sign in, time out, SBAR reporting, checklists” etc. were implemented. Contacts between the team and the referral clinicians were also enhanced and defined in the VSM.
- Coaching and enhancement of team work.
  During the preparatory work and the implementation-process substantial work were done to train awareness and competence in team dynamics and development. Supported by external consultants, this was maintained in recurrent seminars during the study period to strengthen “culture” development and commitment.
- Institution of an Intensive Care fellowship.
  To ensure adequate physician competence, a two-year fellowship in Intensive Care was instituted. This included the clinical training program instituted by the Scandinavian Society for Anaesthesia and Intensive Care (SSAI) and was extended by a mandatory scientific project, a 3 month rotation to a foreign university ICU and a course in personalised leadership and management. Mandatory for graduation as Intensivist was successful examinations for the European Diploma of Intensive Care (EDIC part I and II).
- Institution of full time Intensivist managed care (24 h/7d)
- Fully integrated intensivist and paramedical (physiotherapist, Social counsellor) staff in the nursing team.
  The previous team consisting of CCN and assistant nurses were reinforced with Intensivist and paramedic staff.
- The introduction of multidisciplinary rounds
  All patients were discussed at a daily 30 min conference with participants from all referral specialities as well as from the teams. This provided a concrete opportunity for clinical decision-making, consensus on planning and priorities and for education.
- Repetitive clinical and managerial audits.
  Repetitive audits were conducted in parallel with current monitoring of unit performance. These were done by the ICU management, reinforced by the unit’s economist and HR personnel. The results were available on line for the ICU staff and presented to referral specialities and hospital administration at 4 month intervals.

**Table 1:** Temporal description of the QI process and a description of the components

Main objectives	Phase 1 2006 Trust, values and objectives	Phase 2 2007–2008 Learning organization	Phase 3 2008–2009 Team development	Phase 4 2009 Shared management
Intervention				
Education/ training	2 days training of designated VSM taskforce and nine one day seminars with the whole staff and the creation of workgroups. Follow ups in monthly discussions in the work groups. “Transformal leadership” training with all clinical managers led by external consultants.	Training of the VSM taskforce in methodology, scooping and workflow.	Data from audits and unit performance discussed and processed at one day seminars with all staff. Additional overnight seminar with physicians. Coached by external consultants.	Training of clinical managers in working in a process orientated organisation.
Value Stream map (VSM)		Executed by the VSM taskforce		
Continuous improvement & visual management	Improvement suggestions and projects resulting from the seminars were edited for use in phase 2.	Feedback and results after the implementation were discussed with all staff at four seminars. New improvement tasks were distributed.	Internal and external working environment audits. Reporting of clinical and economical performance.	Internal and external working environment audits. Reporting of of clinical and economical performance.
Work redesign	Institution of fellowships for the education of intensivists.	Outcome of VSM and new workflow presented to all staff at four implementation meetings.	Revision of the previous VSM according to achieved results and feedback from seminars and audits.	
Teamwork	Implementation of “transformal leadership”.	Multiprofessional teams were instituted.	Expansion of the team with paramedic staff. Seminars on individual contribution and group dynamics.	Re-distribution and re- interpreting of formal mandates, leadership and responsibilities.

### Design

Retrospective single-centre quality improvement study.

### Setting

A 12-bed mixed ICU in a 600-bed tertiary teaching hospital in southern Sweden.

Clinical staff: 16 physicians (10 Intensivists, 6 Anaesthetists in intensivist training programs), 90 ICU nurses (CCN), 90 assistant nurses, two physiotherapists and one social counsellor.

### Target population

Adult ICU patients, 18–89 years of age consecutively admitted to the ICU in 2008–2014.

### Target process

We defined the study process as all clinical activities conducted during a 24 h period in the ICU.

### Scientific production management

During the process analysis and revision of the unit’s workflow, scientific production management methodology was used [27–29]. This concept utilises a flow directed approach to process analysis described as “Value Stream Mapping” (VSM), targeting a designated work process. The central methodological issue is to identify the “value adding activities” in the targeted work flow and consequently, to separate these from those not adding value to the process (waste). The waste items are categorised and analysed in 7 separate domains which are [14]: 1) Waiting, 2) Transport, 3) Over processing, 4) Inventory, 5) Motion, 6) Defects, and 7) Talent waste.

The first step in the development of a VSM is to describe all the activities conducted during the defined timeframe and to organise these chronologically. From this chronologically organised workflow, the “value adding activities”, clinical endpoints and “delivery deadlines” are identified. The “present situation” is then concluded by the identification of “waste” items. From this, a revision of the flow is conducted to describe an alternative “desired situation” and the items, actions and methods necessary for a successful revision. During the revision of the present workflow the “waste” identified in the VSM is addressed by a root cause analysis leading to suggested actions for improvement. The process is finally concluded with a plan for implementation (Fig. 1).

### Flow oriented working process

A program for organisational change was developed using the method of scientific production management as described above [19]. The program went fully operational and was introduced into clinical practice in 2008. The change process was driven by a multi-professional task force who received training in scientific production management. At the initiation of the change process, seminars with all staff were conducted where shared values and ethics were discussed and areas for improvement were identified. The task force then performed a VSM analysis of the total ICU 24 h workflow and outlined a new flow- adapted organisation focused on optimising ability and capacity to deliver EBM critical care 24 h/7d. To obtain adequate competence, physician participation and to facilitate teamwork, a two-year program for physician subspecialisation in intensive care (fellowship) was instituted in 2006 and physician staffing was altered from Anaesthetists on rotations and night shifts to full-time, in house, ICU physicians on a 24 h/7d basis by the time when the program went clinically operational in 2008.

Also in 2008, the traditional physician two-shift schedule was abandoned and replaced with a three-shift model where handover times were synchronised with the rest of the team. The number of nursing staff/physicians per shift was set according to the anticipated clinical workload and identified tasks defined in the VSM.

The implementation process was followed up by repetitive audits with all staff throughout the study period. One minor revision of the original VSM concerning round times and the multidisciplinary round was made in the beginning of 2009 in accordance with the audits. Besides from this, no further interventions were done during the study period. The progress of the unit was recorded in terms of patient outcome and economic results. The principal steps of the process are outlined in Table 1.

### Ethical considerations

All clinical data were anonymously collected and there were no possibilities to identify individual patients in the dataset. All treatments were performed in compliance with standard evidence-based clinical practice and at the discretion of the treating physician.

At admittance, all patients and their relatives were informed, verbally and in writing, that during the ICU stay, clinical data were continuously recorded and reported to regional and national ICU databases. This is a standard, nationally agreed procedure for performance and quality monitoring and part of normal ICU routines. The information also pointed out that given consent to this data retrieval was voluntary and could be withdrawn at any time. In that case, all data would be erased.

According to Swedish law, ethical approval is not required for QI–projects and monitoring of normal clinical practice [30].

### Data collection

Clinical performance and outcome data were prospectively collected from the regional ICU database (Patient Administrative System Intensive Care, PASIVA) and the hospital detailed accounting system. Patient characteristics were obtained from the PASIVA system and included age, gender, cause of admittance and Simplified Acute Physiology Score (SAPS III).

The data collection was organised in two specific groups:

- Clinical outcome data
- Health economic data

### Clinical outcome data

Primary outcomes: ICU mortality, 30-day mortality and Kaplan-Meier estimates of mortality with a follow-up period of 20 months after ICU admittance.

Secondary outcomes: Length of ICU stay (LOS) and time on ventilator.

### Health economic evaluation

We estimated the cost of the intervention by listing the number of hours the different categories of personnel worked with implementation or upkeep of the intervention. This information was collected during the intervention. Work hours were valued according to the average cost to the employer for employing a person from each personnel category, operationalised as salary including taxes and social contributions. This information was taken from Statistics Sweden [31] and Ekonomifakta [32]. Data for estimating ICU costs were obtained from the hospital’s detailed accounting system.

All costs are discounted at 3% and presented in the price level of 2014 using the Swedish consumer price index [33]. All costs are presented as Euro using the 2014 exchange rate of €1 = 9.0968 SEK.

The outcome of the intervention was measured in terms of life years gained (LYG) and quality-adjusted life years gained (QALY). For life years gained (LYG), we estimated the age- and gender-specific life expectancy for ICU survivors using the data from the 2010 Swedish life expectancy tables [34]. To obtain net LYG, the amount of yearly LYG was multiplied with the result of the annually calculated Kaplan-Meier estimates of ICU one-year survival. This data was obtained using survival data from the regional ICU database (PASIVA). The quality-adjusted life years of ICU survivors (QALYs) were obtained by multiplying net LYG by a previously established utility weight of 0.73 for ICU survivors [35]. The effects were not discounted.

We estimated the cost-effectiveness of the intervention, from a healthcare perspective, using the situation in 2008 as the alternative in absence of the intervention. The cost of the intervention compared to the incremental gain in life years and QALY over the study period was then calculated. In a sensitivity analysis, we included the incremental cost of the ICU over the study period (excluding the cost of the intervention to avoid double counting).

### Statistical analysis

Descriptive statistics were made with Microsoft Excel (2010) and QlikView (QlikView 11). Student’s t-test was used to compare differences between years regarding length of stay and time on ventilator due to the large sample. Kaplan-Meier was used for analysis of survival. These tests were made with SPSS version 22 (IBM Corp. Released 2013. IBM SPSS Statistics for Windows, Version 22.0. Armonk, NY: IBM Corp). An online Chi-square Calculator (http://www.socscistatistics.com/tests/chisquare/Default2.aspx) was used to analyse differences between years regarding changes in group sizes.

## Results

### Demographics

**Fig. 2:**
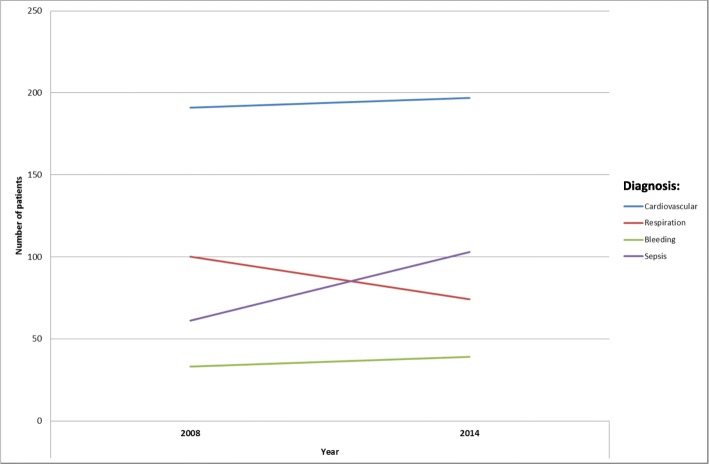
Frequency of diagnoses 2008 and 2014, grouped by major area

During the study period (2008–2014), a total of 5950 patients were included in the follow-up. Over the study period there was a 19% increase in the number of ICU patients with the increase predominantly in patients > 65 years (*p* < 0,05). In the total population, the proportion of female patients increased over the study period (*p* < 0,05). The increase in total caseload was mainly due to a significant increase in septic patients (*p* < 0,01) (Table 2) while the distribution of remaining admitting diagnoses remained unchanged (Fig. 2). The case load severity/patient (SAPS III score/ patient) showed no significant alterations over the study period (Table 2).

### Length of stay (LOS)

During the study period, ICU length of stay (LOS) decreased in the overall population (ns). Among septic patients, there was a significantly reduced LOS (*p* < 0.01) (Table 2).

**Table 2:** Descriptive results of the ICU patients and statistically tested changes

Admittance Year Parameter	2008 n (%)	2009 n (%)	2010 n (%)	2011 n (%)	2012 n (%)	2013 n (%)	2014 n (%)	Change 2008 vs 2014 n (%)	p (n.s. = non significant)
No of patients	768	814	905	847	836	866	914	146 (19.0)	n.s.
0–19 years of age	26 (3.4)	33 (4.1)	30 (3.3)	24 (2.8)	23 (2.8)	25 (2.9)	19 (2.1)	−7 (− 26.9)	n.s.
20–64 years of age	379 (49.3)	367 (45.1)	416 (46.0)	384 (45.3)	340 (40.7)	395 (45.6)	419 (45.8)	40 (10.6)	n.s.
> 65 years of age	363 (47.3)	414 (50.9)	459 (50.7)	439 (51.8)	473 (56.6)	446 (51.5)	476 (52.1)	113 (31.1)	< 0.05
Female	282 (37.7)	331 (40.7)	383 (42.3)	366 (43.2)	361 (43.2)	352 (40.6)	382 (41.8)	100 (35.5)	< 0.05
Septic diagnosis n(%)	61 (7.9)	79 (9.7)	69 (7.6)	76 (9.0)	82 (9.8)	103 (11.9)	103 (11.3)	42 (68.9)	< 0.01
Case Severity (SAPS III/patient)	34.2	32.3	31.7	33.4	33.5	31.3	32.6	−1.6 (−4.7)	n.s.
Septic patients	56.6	57.1	56.3	57.5	56.8	51.1	56.4	−0.2 (−0.4)	n.s.
Length of stay (LOS) average days	3.2	3.1	2.8	3.3	2.8	2.9	2.8	−0.4 (−12.5)	n.s.
Septic patients	7.9	5.7	5.8	5.7	5.8	4.0	4.5	−3.4 (−43.0)	< 0.01
Ventilator treatment, n of patients	467 (60.8)	472 (58.0)	519 (57.3)	517 (61.0)	524 (62.7)	565 (65.2)	591 (64.7)	124 (26.6)	n.s.
20–65 years of age	218 (57.5)	206 (56.1)	230 (55.3)	223 (58.1)	204 (60.0)	254 (64.3)	277 (66.1)	59 (27.1)	< 0.05
Time on ventilator (average days)	2.2	1.9	1.7	2.2	1.8	1.8	1.7	−0.5 (−22.7)	< 0.01
Septic patients	6.4	4.0	4.3	4.0	4.6	2.8	3.1	−3.3 (−51.6)	< 0.01
Mortality									
ICU	90 (11.7)	89 (10.9)	91 (10.1)	82 (9.7)	86 (10.3)^a^	75 (8.7)	68 (7.4)	−22 (−24.4)	< 0.01
> 65 years of age	61 (16.8)	63 (15.2)	63 (13.7)	57 (13.0)	57 (12.1)^a^	50 (11.2)	43 (9.0)	−18 (−29.5)	< 0.01
30-days	167 (21.7)	172 (21.1)	188 (20.8)	177 (20.9)	186 (22.2)^a^	168 (19.4)	182 (19.9)	15 (9.0)	n.s.

^a^ Data during this period are influenced by a temporary relocation of the ICU services

### Time on ventilator

There was an increase in the number of ventilator treated patients over the study period with a predominance for the age group 20–65 (*p* < 0.05), who also contributed the most to the overall reduction in ventilator time. However, during the study period, there was a significant reduction in ventilator time in the total population (*p* < 0.01). Among the septic population, this reduction in ventilator time was even more pronounced (*p* < 0.01) (Table 2).

### Mortality

During the period 2008–2014, there was a decreased ICU mortality in the total patient population (*p* < 0.01) and almost a 50% reduction in the subgroup of patients more than 65 years of age (*p* < 0.01). 30d mortality decreased slightly during the study period (ns) (Table 2).

**Fig. 3:**
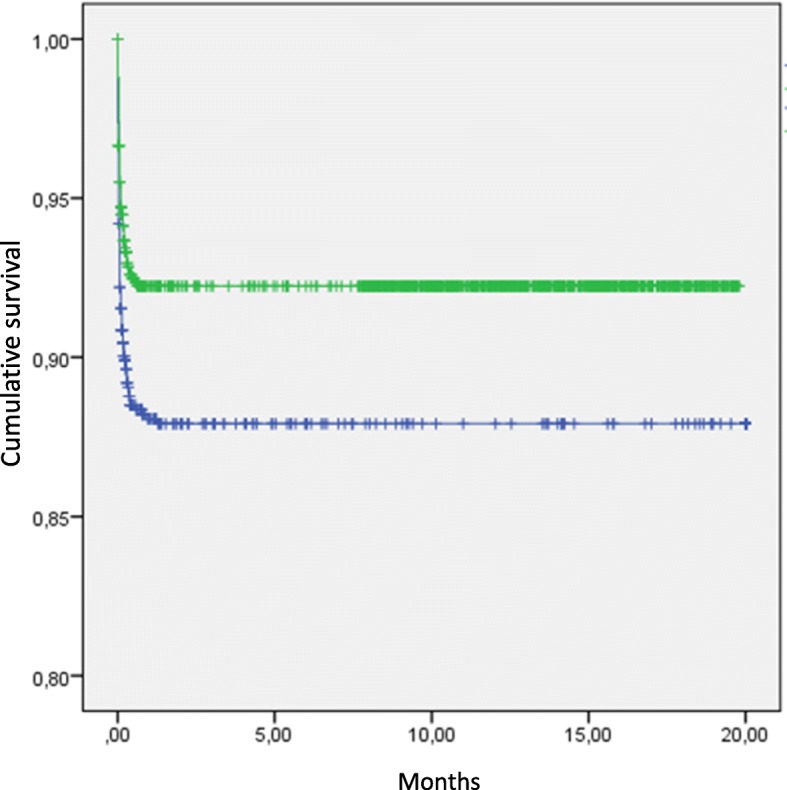
Cumulative survival (y-axis; 0–1) in months (x-axis) 2008 and 2014

Extended follow-up over 20 months (600d) showed a remaining improved survival in the total population (*p* < 0,003) 2008–2014 (Fig. 3).

#### Health economics

During the study period (2008–2014), the net yearly ICU cost remained rather stable although with a downward trend (€12.9 million +/− 1.0 in the price level of 2014, see Table 3). The cost of the intervention over the study period amounted to €0.7 million (Table 4). Regarding costs, 45% of the cost is related to preparatory work previous to the intervention in 2006–2008 and the remaining related to intervention upkeep 2009–2014 (Tables 1, 4). The average yearly cost of upkeep sums to €66,373. The number of LYG and QALY, produced during the study period, increased by 21%, respectively, compared to 2008. This resulted in an incremental effectiveness of the intervention of 9600 QALY over the study period (Table 4). The cost-effectiveness ratio thus sums to €75 per QALY gained or €55 per life year gained (Table 5). If we also include the cost of the ICU and not only the cost of the intervention, the intervention is found to be both cheaper and better in terms of number of QALY produced compared to 2008, i.e. the intervention is dominating.

**Table 3:** Total ICU cost excluding cost related to the intervention (€)

Year	ICU cost (millions)
2007	13.9
2008	12.9
2009	13.5
2010	12.0
2011	12.2
2012	13.0
2013	13.4
2014	12.1

**Table 4:** Cost and effect of the intervention

	2006	2007	2008	2009	2010	2011	2012	2013	2014	Total
Cost (€)	141,682	143,334	39,989	95,568	41,276	90,946	93,246	38,600	38600^a^	723,241
LYG			15,947	16,879	18,878	17,753	16784^b^	18,393	20,133	
Incremental LYG cmp to 2008				932	2931	1807	838^b^	2447	4186	13,140
QALY			11,641	12,322	13,781	12,960	12253^b^	13,427	14,697	
Incremental QALY cmp to 2008				681	2140	1319	611^b^	1786	3056	9593

^a^ The maintenance cost for 2014 is assumed to be the same as for 2013

^b^ Data during this period are influenced by a temporary relocation of the ICU services

**Table 5:** Cost-effectiveness of the intervention (€)

	Excluding ICU cost	Including ICU cost
Cost/QALY 2008–2014	75	Intervention dominates
Cost/life year 2008–2014	55	Intervention dominates

## Discussion

In this paper, we show enhanced ICU performance and outcome after implementation of a quality improvement program in a university general ICU.

ICU-mortality was significantly reduced for all age groups. The survival benefit seemed to have been established during the time in the ICU, since both 30-d mortality and long term outcome only showed minor further reductions after discharge from the ICU (Fig. 3). We suggest that this observation could be interpreted as a possible beneficial effect of the interventions. The beneficial effects were most pronounced in septic patients and in the expanding patient group > 65 years of age. These groups made up most of the increase in caseload and also seemed to have benefited the most in terms of improved outcome, time on ventilator and LOS.

With regards to the characteristics of the interventions, it is impossible to appreciate the single value or impact of each item. Therefore, the results should be evaluated in the light of the combined efforts. However, improvements in patient outcome and cost-effectiveness were progressively increasing over the study period and in parallel with the interventions made. The findings of our study are in line with the results from previous studies where introduction of intensivist staffing, development of multi-professional teams and more thorough process control have been shown beneficial [4, 19, 36].

Significant interventions in our study were to implement intensivist staffing 24 h/7d, to organise staff members into multi-professional teams and to synchronise working hours, intra-disciplinary collaboration and assignments within the teams. These efforts comply with the existing body of research, indicating that efforts to ensure timely interventions, adherence to EBM guidelines and clinical process control have a significant impact on clinical outcomes [2, 14, 25, 36, 37]. Organising the workforce in teams has also previously been found to contribute to the improvement of clinical performance, enhance the appreciation of shared goals and values and improve the working environment [4, 16, 21, 23, 26].

Intensive care consumes substantial resources and its costs are increasing [1, 6, 14, 24, 38]. Interventions in the critical care services are likely to generate economic results further along in the healthcare chain rather than during the stay in the ICU. Therefore, improvement undertakings, like the ones described in this paper, should be executed in a cost-effective manner to motivate implementation. In our study, we were able to show an increase in produced QALY and LYG paralleled by a subsequent cost reduction during the observation period. The improved outcome and, in particular, the outcome results in the elderly and septic patient groups paralleled with reduced ICU costs and mortality could thus motivate the investment connected to improvement projects in critical care.

If we include the effect on the cost of running the ICU in the cost-effectiveness calculations, the intervention is found to be dominating as it is both cheaper and better in terms of gained QALY compared to the alternative treatment situation. However, as it is difficult to establish that the full reduction in ICU costs are due to the intervention, our base case results take a conservative approach and exclude this possibility altogether. The intervention is then found to be very cost-effective, with a cost per QALY of €75. Although establishing an exact valuation of a QALY is difficult, €75 per QALY is, by any standard, extremely cost-effective. A cost per QALY below €11,000 has been considered to constitute a low cost in Sweden (i.e., to be highly cost-effective) [39].

These results comply with previous studies where improved outcomes reduced incidence of complications and enhanced resource utilisation have been related to improved process control and enhanced adherence to EBM guidelines in a cost-effective manner [6, 10, 11, 13, 40].

### Strengths and weaknesses

As our intervention consisted of a series of composite actions, it is difficult to value the contribution of one single intervention in relation to the achieved results. The study describes a series of quality improvement interventions in a single site and was not designed for benchmark against other units. Because of this, and the anticipated difficulties in finding a well matched control site with identical case mix and organisation, all comparisons were done against own data generated during the study period with the 2008 outcome data serving as baseline.

All data were generated in the normal process of unit performance monitoring. Patient management was performed according to current accepted EBM procedures and at the discretion of the treating physician, who was not involved in the follow-up or briefed on the results.

The single centre design might influence the generalizability in a broader perspective or when applied in a different setting. However, our interpretation of the results is supported by recent systematic reviews and reports. These have emphasised the positive role on improved outcome as an effect of performance improvement programs on adherence to treatment guidelines, and enhanced capacity of performing early appropriate interventions [14, 41, 42].

The follow up period was long and one might argue that the clinical setting might have changed considerably during that time. However, with the exception of an increasing number of patients > 65 years of age and an increase in septic admissions, which rather would have been expected to promote a detrimental trajectory, the clinical characteristics and severity score (SAPS III) in the total sample showed no significant variation between the years indicating a stable case-mix. Likewise, all other organisational, clinical and staffing parameters remained unchanged during the follow up period and thus we suggest that the progressive improvements in performance and outcome seen during the study should be considered as a result of the intervention.

During the last 9 months of 2011 the ICU was relocated to a temporary facility. During that time, normal operations were moderately restricted due to inadequate facilities. Follow up on the patients admitted during that time showed a negative development in clinical outcome, a phenomenon previously reported by others in connection with ICU relocations [9]. Thus, the ICU outcome figures for 2011 / 2012 might have been negatively influenced which should be considered in the interpretation of the temporal development in costs and mortality. However, this temporary loss of performance, in our opinion, strengthens the previous discussion, as the results seems to be highly sensitive to organisational compliance.

Given the design of the study, we cannot be completely confident that the effects on health economics found over time were fully due to the intervention. We therefore also calculated the minimum number of QALY required to be attributed to the intervention in order for the intervention to remain highly cost-effective (using the Swedish valuation of €11,000). Only 66 QALY, or 0.7% of the observed QALY gain, needed to be due to the intervention for it to be highly cost-effective. Alternatively, given the estimated gain in QALY, the cost of the intervention could be almost €105 million and still remain highly cost-effective (to be compared to the actual cost of € 0.7 million). We therefore find it convincing that the intervention has been cost-effective from a healthcare perspective and thus well worth implementing.

## Conclusion

In this single centre study, we have shown favourable outcome results of a QI project aiming to improve the clinical performance and quality through the implementation of multi-professional interaction, teamwork and revisions of work processes. The economic evaluation shows that the intervention is highly cost-effective, a conclusion that is robust in relation to uncertainties concerning costs and effects.

To further establish the value of the components of enhanced process control in the ICU and enhance generalisability of the results of this study, we suggest further and more extensive research in this field, preferably in a prospective multicentre controlled design, preferably with special focus on septic and elderly ICU patients.
